# Jean-Martin Charcot at 200: revolutionizing neurology through a multidisciplinary lens

**DOI:** 10.1055/s-0045-1813236

**Published:** 2025-12-22

**Authors:** Marleide da Mota Gomes, Marcos Raimundo Gomes de Freitas

**Affiliations:** 1 Universidade Federal do Rio de Janeiro, Faculdade de Medicina, Departamento de Medicina Interna, Programa de Pós-Graduação em Psiquiatria e Doenças Mentais, Rio de Janeiro RJ, Brazil.; 2 Universidade Federal do Rio de Janeiro, Instituto de Psiquiatria e Instituto de Neurologia, Laboratório de História da Psiquiatria, Neurologia e Saúde Mental, Rio de Janeiro RJ, Brazil.; 3 Universidade Federal Fluminense, Faculdade de Medicina, Departamento de Medicina Interna, Niteroi RJ, Brazil.

**Keywords:** Jean-Martin Charcot, History of Medicine, Neurology, Neuropsychiatry, Teaching, Neurodegenerative Diseases, Hysteria, Hypnosis, Art

## Abstract

As we near the bicentenary of his birth, Jean-Martin Charcot (1825–1893) is remembered not only as the founder of modern neurology but also as a *uomo universale* . His multidisciplinary approach transcended 19 ^th^ -century medicine, establishing neurology as a distinct discipline while integrating art, psychology, and philosophy into his study of the nervous system. His work laid foundations for neurodegenerative diseases (amyotrophic lateral sclerosis [ALS], Parkinson's disease, multiple sclerosis [MS]), functional neurological disorders (FNDs), and psychoanalysis, foreshadowing neuroplasticity and the mind-body connection. His innovative teaching at Salpêtrière—merging anatomy with artistic documentation—revolutionized medical education, inspiring figures from Freud to modern neuroscientists. Two centuries later, Charcot's legacy endures not just in eponyms but in his unifying vision of brain, mind, and art - a timeless model for interdisciplinary medicine. The present paper explores his impact on neurodegenerative research, functional disorders, medical pedagogy, and the humanities.

## INTRODUCTION

Few figures in medical history have left as enduring an impact as Jean-Martin Charcot. Born in Paris in 1825, he rose from modest origins to become the leading neurologist of his time ( Table 1 ). Marking his bicentenary, the present article revisits Charcot's integrative vision – spanning neurology, psychiatry, teaching, and the arts – and reflects on its relevance for medicine today. 1 2 3

**Table 1:** Jean-Martin Charcot (1825 - 1893): Founder of Modern Neurology – Biographical and Professional Summary 1 2 3 4 5 6 7 8 9 10 11

Category	Details	Notes
**Birth & death**	Born November 29, 1825 (Paris); died August 16, 1893 (Lac des Settons, France); buried at Montmartre Cemetery.	Died of acute pulmonary edema (possibly heart failure). Buried with Catholic rites (unusual for his secular views).
**Family**	Parents: Simon-Pierre Charcot (coach builder) & Jeanne Saussier; eldest of four children.	Artisan family background influenced his meticulous methods.
**Marriage & children**	Married Augustine-Victoire Laurent (1864; her dowry funded his work). Children: Jeanne, Jean-Baptiste (renowned Antarctic explorer), and stepdaughter Marie Durvis.	Wife's wealth enabled his research collections; son extended the Charcot legacy in exploration.
**Education**	BA in Letters (Sorbonne, 1843); MD, University of Paris (1853). Thesis: *“Études pour servir à l'histoire des affections articulaires”* (self-illustrated study of gout/rheumatism).	Early focus on joint pathology; transitioned to neurology under Pierre Rayer, with key mentorship from Guillaume Duchenne.
**Key Positions**	Salpêtrière Hospital (1848 - 1893; director from 1862); Professor of Pathological Anatomy (1872); The first dedicated professorship in neurology (1882).	Transformed Salpêtrière into the global epicenter of neurology.
**Major clinical description/contribution**	ALS (“Charcot's Disease”), MS (Charcot's Triad), Parkinson's pathology, Charcot joint (neurogenic arthropathy), hysteria as a neurological disorder.	Defined neurodegenerative diseases and psychosomatic conditions; later theories debated. **Criticism:** Theatrical hysteria demonstrations (“ *Grand Hystérie* ”) later criticized as exploitative.
**Methods**	Clinicopathological correlation, hypnosis, ophthalmoscopy, vibration therapy.	Bridged neurology and psychology; pioneered psychoneurology.
**Notable Disciples**	Babinski, Tourette, Janet, Freud; 33+ interns (incl. Bechterew, Marinescu).	Influenced neurology and psychoanalysis; depicted in *Leçon Clinique* (1887). **Art Note:** Medical illustrations used in Surrealist art; influenced Freud's *Studies on Hysteria* .
**Chair Successors**	Brissaud (1893–94), Raymond (1894–1910), Déjerine (1910–17), Pierre Marie (1917–24).	Maintained Salpêtrière's dominance in neurology.
**Eponyms**	**Neurology:** Charcot's Disease (ALS), CMT (Charcot-Marie-Tooth), Charcot-Bouchard aneurysms, Charcot's Triad (MS). **Internal Medicine:** Charcot-Leyden crystals, Charcot-Wilbrand syndrome, Charcot's intermittent claudication, Charcot's Triad (acute cholangitis). **Rheumatology:** Charcot joint. **Psychology:** Charcot's zones (hysteria).	12+ eponyms across disciplines; legacy in neurology, pathology, and rheumatology.
**Legacy**	Founded the Salpêtrière School; merged art and medicine (commissioned illustrations); standardized neurological exams; reviewed 600+ theses.	Influenced medical education, Surrealism (e.g., André Breton), and interdisciplinary science.
**Personal Traits**	Fluent in 5 languages; enthusiast of Shakespeare, classical music, and academic painting. Austere yet magnetic lecturer. Skeptical of germ theory; emphasized hereditary pathology (pre-genetics).	Though aesthetically traditional, revolutionized neurological illustration. Complex legacy: controversial yet transformative.

## NEURODEGENERATIVE DISEASE RESEARCH: CHARCOT'S LASTING INFLUENCE

At the Salpêtrière, Charcot created a hub of neurological innovation, blending clinical observation, pathological anatomy, and dynamic teaching.

Charcot's work on neurodegenerative diseases began at the Hôpital de la Salpêtrière – once a neglected asylum, where he collaborated with Alfred Vulpian. 1 Together, they conducted an extensive inventory of the vast patient population, mostly elderly women. As of July 1, 1862, the year they took over, there were 2,635 residents classified as “indigents and non-insane epileptics.” Charcot and Vulpian discovered that many of these women suffered from chronic disorders of the nervous, musculoskeletal, and sensory systems. However, the rudimentary state of medical knowledge at the time hindered precise diagnoses. Boumeville later referred to their extensive documentation as the *Archives Médicales of the Salpêtrière* .

The foundation of functional neurological disorder (FND) studies and the origin of psychoanalysis also come from this place.

In 1866, Charcot and Vulpian defined multiple sclerosis (MS) through clinicopathological studies, identifying demyelinating lesions and preserved axons. 4 His 1868 lectures further classified MS into cerebral, spinal, and mixed subtypes.

These early insights into demyelinating disease laid the groundwork for Charcot's subsequent work on other neurodegenerative conditions, including amyotrophic lateral sclerosis (ALS).

In 1874, Charcot connected ALS to degeneration of the lateral spinal cord and anterior horn cells, unifying its clinical and pathological features. 5 He was the first to recognize ALS as a distinct disease.

Charcot also redefined Parkinson's disease, highlighting rigidity and bradykinesia alongside tremor, and hypothesized a subcortical origin—insights that align with current understanding of basal ganglia involvement. 3

## FUNCTIONAL NEUROLOGICAL DISORDERS, HYPNOSIS, AND THE BIRTH OF PSYCHOANALYSIS

Jean-Martin Charcot redefined hysteria, now known as FND, by proposing functional brain disturbances without structural damage. His concept of dynamic lesions anticipated modern neuroscience's understanding of network-based dysfunction. 6 7 At the Salpêtrière, Charcot combined rigorous clinical observation with hypnosis, using it both diagnostically and therapeutically within a neurological framework.

At the Salpêtrière, he merged rigorous clinical observation with hypnosis, using it diagnostically and therapeutically within a neurological framework.

Charcot's classification of *la grande hystérie* into four phases established the first nosology for what would become conversion disorder. His dramatic demonstrations—immortalized in André Brouillet's *A Clinical Lesson at the Salpêtrière* —drew global attention (and criticism for potential suggestibility). 8 Among the attendees was Sigmund Freud, whose 1885 to 1886 observership under Charcot proved transformative. Freud's 1893 obituary credited Charcot with shifting his focus from neuropathology to psychopathology, 9 directly inspiring Freud's theories of unconscious processes and repression.

Beyond hysteria, Charcot's anatomical-clinical method revolutionized neurology. He identified and characterized conditions like tabes dorsalis, multiple sclerosis, and ALS. His *Leçons du mardi* —charismatic Tuesday lectures—blended spontaneous case discussions with structured teaching, fostering an international academic network.

In the 1880s, Charcot legitimized hysteria as a medical subject, distinguishing it from organic disease and demonstrating its psychological underpinnings. His hypnotism studies revealed how subconscious ideas manifest physically, influencing Pierre Janet, Josef Breuer, and Freud. While later critiques targeted his heredity focus and hypnosis classifications, modern neuroimaging validates his insights: FND involves disrupted networks for emotion (anterior cingulate-insula-amygdala), agency (temporoparietal junction), and predictive coding (frontoparietal circuits). 7 Contemporary treatments (e.g., cognitive-behavioral therapy, neuromodulation) still reflect Charcot's integrative neurology-psychology model.

Charcot's legacy endures not only in clinical practice but also in broader cultural narratives. By merging clinical observation with artistic documentation (e.g., Salpêtrière's iconography), he bridged science and creativity. His fundamental questions about mind-brain interaction remain central to neuropsychiatry, with Freud standing as his most prominent intellectual heir.

## CHARCOT AS EDUCATOR AND ARTIST: THE MULTIFACETED ARCHITECT OF MODERN NEUROLOGY

At the Salpêtrière, Jean-Martin Charcot cultivated a dual legacy as both a transformative educator and a visionary artist-neurologist. His famed *Leçons du mardi* (Tuesday Lectures) became an intellectual crossroads, attracting luminaries such as Freud, Babinski, and Gilles de la Tourette, who would later shape their own disciplines. 10 Through his rigorous clinical methodology—pairing meticulous observation with systematic nosology—Charcot not only laid the foundations of modern neurology but also redefined medical pedagogy itself. Beyond the lecture hall, he wielded significant influence in academic governance, serving on doctoral juries and nurturing nascent theories, particularly in his groundbreaking work on hysteria. 11

**Figure 1:**
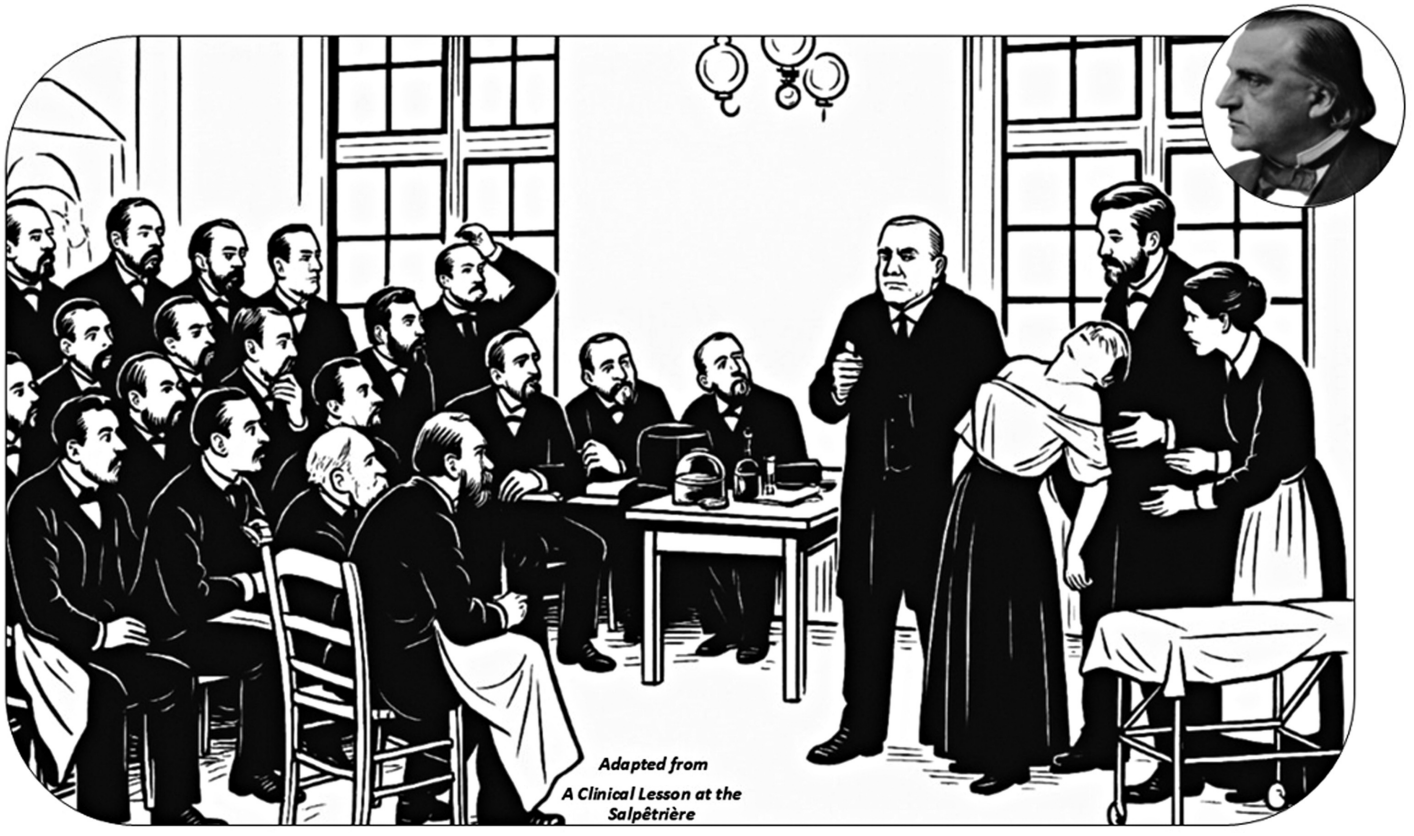
A contemporary remake of *Une Leçon clinique à La Salpêtrière* (1887) by André Brouillet, created through digital interpretation, serves as both a tribute to Jean-Martin Charcot and a vivid portrayal of the scientific prestige of 19 ^th^ -century French medicine. 10 The composition reconstructs the Salpêtrière Hospital amphitheater setting of Charcot's famous Tuesday lecture on hysteria, featuring hypnosis as a diagnostic and pedagogical tool. At center stage, Charcot presents Blanche Wittman in grande hystérie, supported by his assistant Joseph Babinski, with head nurse Bottard observing. The audience includes many key figures in neurology and psychiatry, reflecting the intellectual vitality of the moment. Among them are Georges Gilles de la Tourette, Pierre Marie, Charles Féré, and Désiré-Magloire Bourneville. Paul Richer, anatomist and artist, appears beside Charcot. Also present are R. Vigouroux, H. Parinaud, H. Berbez, A. Londe (medical photographer), G. Guinon, L. le Bas, A. Gombaut, A. Arène, J. Claretie, A. Naquet, and G. Bellet. In the background are V. Cornil, P. Burty, M. Debove, M. Durval, and Jean-Baptiste Charcot, the professor's son and future polar explorer, along with P. Berbez, E. Brissaud, and A. Joffroy.

Charcot's genius lay in his synthesis of art and science. The iconic painting *Une Leçon Clinique à La Salpêtrière* ( Figure 1 ) epitomizes this fusion, capturing both the theatricality of his demonstrations and their scientific precision—a tableau vivant of neurology's emergence as a distinct field. His approach transcended traditional boundaries: sketches, photographs, and even performative lectures became tools for medical instruction. This interdisciplinary ethos extended beyond medicine; works like *Les Démoniaques dans l'art* 12 positioned him as a cultural touchstone, bridging clinical detail with artistic expression and foreshadowing Surrealism's fascination with the unconscious.

**Figure 2:**
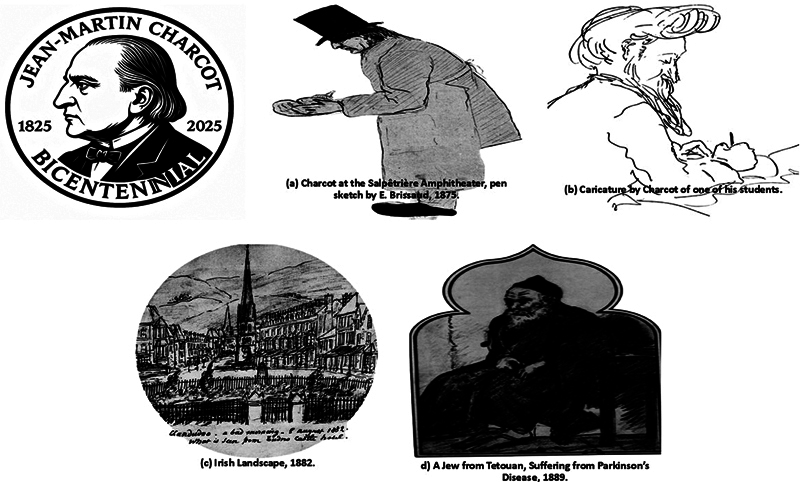
Depicting the Mind of a Master: Charcot's Drawings, His Iconography, and a Bicentenary Homage. Drawings made by (b - d) or of Charcot (a). 15 The figure also includes a commemorative medal honoring the bicentenary of Charcot's birth.

As an artist, Charcot was equally pioneering. Collaborating with Bourneville and Regnard (1876–1880), he produced the *Iconographie photographique de la Salpêtrière* , 13 a seminal visual archive of hysteria that set new standards for medical documentation. 14 His sketches—such as *A Jew from Tetouan, Suffering from Parkinson's Disease* —revealed an uncommon ability to distill both pathology and humanity, uncovering subtle motor phenomena overlooked by contemporaries. 15 This dual identity as clinician and artist ( Figure 2 ) enabled him to perceive patterns invisible to others, transforming observation into revelation. 16

Today, Charcot's visual legacy endures. His photographic techniques remain benchmarks in medical illustration, while his pedagogical innovations continue to inspire clinicians and artists alike. More than a chronicler of disease, he was a cartographer of the mind's intersection with the body—a testament to the enduring power of interdisciplinary vision.

## A *UOMO UNIVERSALE* FOR THE FUTURE

Two centuries after his birth, Charcot's legacy transcends eponyms—it lives on in his revolutionary integration of brain, mind, and art, a paradigm that continues to shape modern interdisciplinary neuroscience. By bridging neuroanatomy, psychology, and artistic expression, he forged a holistic vision of medicine, one that harmonized scientific rigor with clinical intuition. In today's era of artificial intelligence and data-driven medicine, Charcot's approach serves as a vital reminder that technological advancement must be guided by clinical wisdom, human observation, and the irreplaceable art of healing. 17
